# Insights into the role of *Streptococcus oralis* as an opportunistic pathogen in infectious diseases

**DOI:** 10.3389/fcimb.2024.1480961

**Published:** 2024-11-04

**Authors:** Jingyi Ren, Peng Sun, Meijuan Wang, Wenjuan Zhou, Zhonghao Liu

**Affiliations:** ^1^ School of Stomatology, Binzhou Medical University, Yantai, China; ^2^ Department of Implantology, The Affiliated Yantai Stomatological Hospital, Binzhou Medical University, Yantai, China; ^3^ Department of Spine Surgery, 970 Hospital of the People’s Liberation Army Joint Logistics Support Force (PLA JLSF), Yantai, China; ^4^ Yantai Engineering Research Center for Digital Technology of Stomatology, Yantai, China; ^5^ Characteristic Laboratories of Colleges and Universities in Shandong Province for Digital Stomatology, Yantai, China

**Keywords:** *Streptococcus oralis*, opportunistic pathogen, infectious disease, bloodstream infection, infective endocarditis

## Abstract

*Streptococcus oralis*, belonging to the viridans group streptococci (VGS), has been considered a member of normal flora mainly inhabiting the oral cavity. However, more recently, there has been growing recognition of its role as a causative agent in various life-threatening infectious diseases such as infective endocarditis (IE) and meningitis. Additionally, the differences in the prevalence, clinical features, and prognosis of opportunistic infections between *S. oralis* and other VGS species have been addressed. Particularly the predominance of *S. oralis* in IE has drawn critical attention. In potentially fatal infections, clinical neglect of *S. oralis* as an instigating agent might significantly impede early diagnosis and treatment. Nevertheless, to date, the infectious diseases associated with *S. oralis* have not yet been comprehensively described. Therefore, this review will give an overview of infectious diseases caused by *S. oralis* to uncover its hidden role as an opportunistic pathogen.

## 1 Introduction

*S. oralis*, a Gram-positive, nonmotile, alpha-hemolytic bacterium belonging to the *Streptococcus mitis* group, is a member of the viridans group streptococci (VGS). *S. oralis* comprises three subspecies including *S. oralis* subsp. *oralis*, *S. oralis* subsp. *tigurinus*, and *S. oralis* subsp. *dentisani* (Jensen et al., 2016). It has been considered a commensal colonizing the oral cavity, oropharyngeal, nasal, gastrointestinal, and genitourinary tracts with relatively low pathogenicity and virulence. Especially in the human oral cavity, *S. oralis*, an early colonizer of dental plaque, is one of the most abundant commensal microbiota (Li et al., 2004). In addition to humans, it has also been recognized in the commensal flora of higher primates such as great apes (Denapaite et al., 2016). However, recent studies have characterized its potential to instigate severe infections such as infective endocarditis (IE) and meningitis under specific circumstances (Chang et al., 2002). For example, in immunocompromised patients who have undergone oral interventions or with poor oral hygiene, there is a high risk for the organisms to invade sterile body sites and lead to infectious diseases (Cruz Cardoso et al., 2021).

Nonetheless, the poor assignment of this species results in an underestimation of the opportunistic infections caused by *S. oralis*. The taxonomy within VGS species, especially the differentiation between *Streptococcus mitis* and *S. oralis*, has been problematic for decades. Given the fact that *S. mitis* and *S. oralis* share highly identical 16S rRNA sequences (over 99%), common clinical diagnosis techniques including matrix-assisted laser desorption ionization-time of flight mass spectrometry (MALDI-TOF MS), VI-TEK^®^ 2 system, and API^®^ rapid ID 32 Strep system only provide general assignments of limited VGS species but fail to accurately and reliably discriminate *S. mitis* and *S. oralis* (Suzuki et al., 2005; Teles et al., 2011; Isaksson et al., 2015). In recent years, the genotypic sequence analysis of specific genes such as the *rgg* gene (Park et al., 2010), the *sodA* gene (Teles et al., 2011), and the *rnpB* gene (Isaksson et al., 2015) has been proposed to discriminate *S. mitis* and *S. oralis*. Especially multilocus sequence analysis (MLSA) of seven housekeeping genes (*map*, *pfl*, *ppaC*, *pyk*, *rpoB*, *sodA*, and *tuf*) has been applied in prior research to provide relatively satisfactory identification within *S. mitis* and *S. oralis* (Shelburne et al., 2014; Rasmussen et al., 2016; Imai et al., 2020; Jensen et al., 2021). Nevertheless, this method does not seem feasible for clinical utilization in most laboratories since it is time-consuming (Imai et al., 2020; Menon T, 2023).

**Figure 1:**
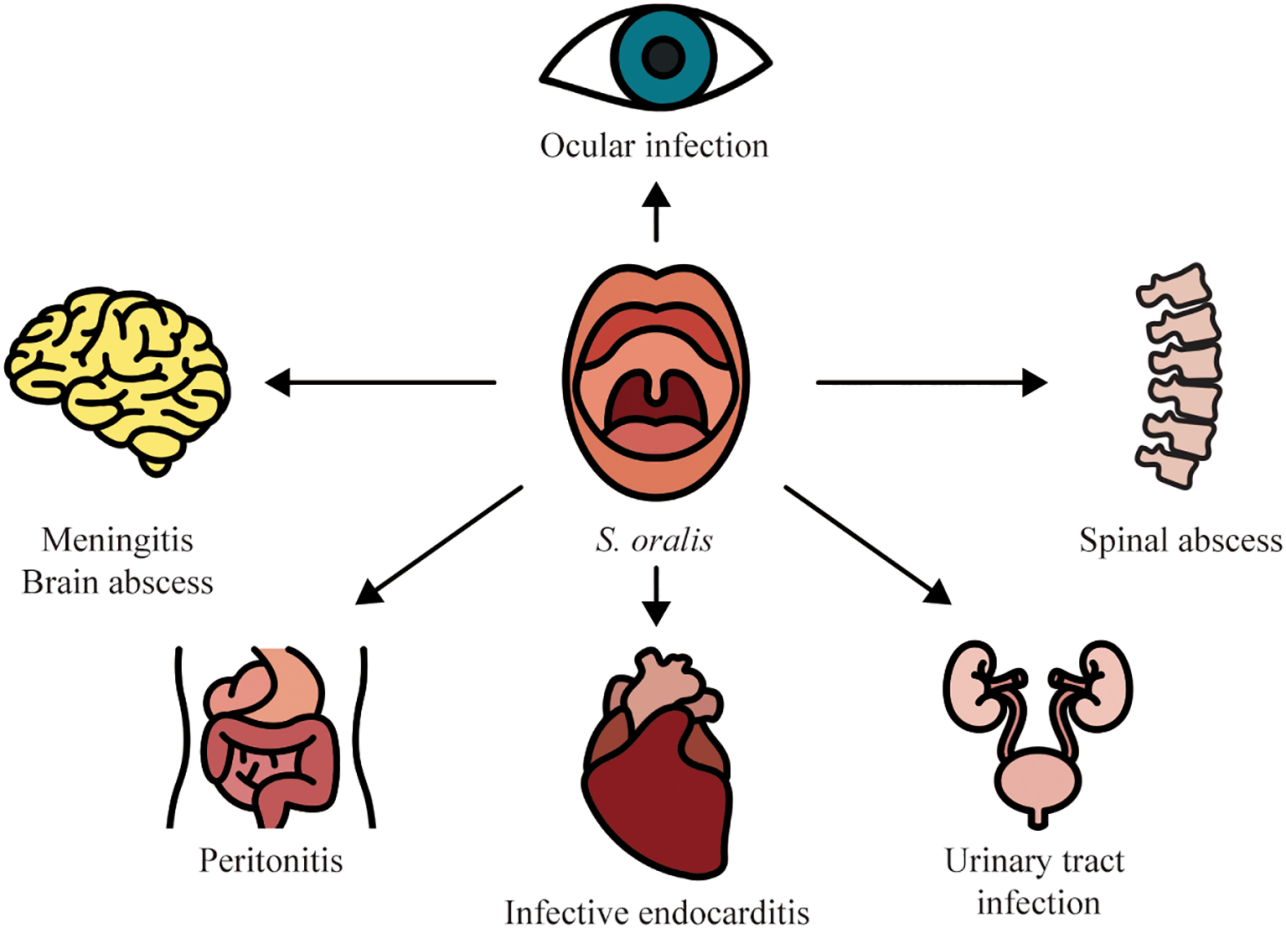
Infectious diseases associated with *S. oralis*.

With the advancement of species-level assignment, the great variations in the distribution in infectious diseases, clinical features, antimicrobial susceptibility patterns, and prognosis among VGS species have been revealed (Chun et al., 2015; Kim et al., 2018; Chamat-Hedemand et al., 2023b). For example, a predominance of *S. oralis* has been recognized in several streptococcal infections such as IE and ocular infections. Identification of *S. oralis* within VGS is of utmost importance for the expedited diagnosis and optimization of antimicrobial therapy. In this review, we sought to summarize the infectious diseases caused by *S. oralis* to offer important insights into its role as an opportunistic pathogen (**Figure 1**).

## 2 Opportunistic infections caused by *S. oralis*

### 2.1 Bloodstream infection

Invasive dental interventions such as tooth extraction and scaling or even daily oral hygiene practices such as tooth brushing and flossing can induce the spread of *S. oralis* from the oral cavity to the bloodstream leading to transient bacteremia (Yumoto et al., 2019). On the one hand, previous research has demonstrated that *S. oralis* is one of the most prevalent causes of streptococcal BSIs, particularly in neutropenic patients (Kennedy et al., 2000). A cohort study including 118 consecutive VGS BSI cases during the period from July 1, 2011, to December 1, 2012, found that *S. oralis* was the second leading cause (22 out of 118) following *S. mitis* (68 out of 118) (Shelburne et al., 2014). Furthermore, this study observed that approximately 80% of the patients had neutropenia and hematologic malignancies. It suggests that neutropenia and hematologic malignancies are important risk factors in VGS BSIs. Additionally, BSIs caused by *S. oralis* have also been reported in pediatric patients. A retrospective case-control study found that 53 BSI cases were caused by *S. mitis*/*oralis* in pediatric patients from January 2015 to March 2017 (Basaranoglu et al., 2019). In agreement with prior findings in adult cases (average age 50) (Shelburne et al., 2014), 34% of pediatric patients with *S. mitis*/*oralis* BSIs also presented febrile neutropenia. Likewise, another retrospective study involving 40 episodes of VGS bacteremia in 38 pediatric patients on chemotherapy for cancer also revealed *S. oralis* was the third leading causative pathogen accounting for 12.5% of isolates following *S. mitis* (55%) and *Streptococcus sanguinis* (25%) (Ahmed et al., 2003). In addition, Kennedy et al. (2003) reported one *S. oralis* BSI case in a pediatric neutropenic patient presenting severe gingivitis indicating the oral entry of *S. oralis* into the bloodstream. However, our understanding of why *S. oralis* BSI is related to neutropenia and hematologic malignancies is notably underdeveloped. Although *S. oralis* BSIs in both adult and pediatric patients generally present favorable clinical outcomes (Shelburne et al., 2014; Basaranoglu et al., 2019), multidrug resistance in *S. oralis* has gradually emerged imposing challenges to the therapy for bacteremia in neutropenic patients (Watanabe et al., 2020). Unfortunately, previous work largely failed to differentiate *S. mitis* and *S. oralis* (Basaranoglu et al., 2019). The epidemiology of *S. oralis* needs to be further determined.

Moreover, there is a significant relationship between *S. oralis* BSIs and IE. It has been established that non-beta-hemolytic streptococcal BSIs are the dominant cause of IE accounting for 13–44% of all cases (Sunnerhagen et al., 2018). In streptococcal BSIs, *S. oralis* presents a higher IE risk in comparison with other streptococcal species. Chamat-Hedemand et al. (2020) evaluated the risk of IE in 6,506 streptococcal BSI cases in the capital region of Denmark from 2008 to 2017. The author observed the highest IE prevalence in *S. mitis*/*oralis* BSIs (19.4%) compared with a variety of streptococcal species including *Streptococcus pneumoniae*, *Streptococcus pyogenes*, *Streptococcus anginosus*, and *Streptococcus salivariu*. Moreover, the same author revealed that one-year mortality was higher in the patients with *S. mitis*/*oralis* BSIs (Chamat-Hedemand et al., 2023b). It highlights the importance and necessity of species-level identification within streptococcal species for the prediction of prognosis in patients with streptococcal BSIs. Consistently, a retrospective cohort study in South Korea, involving 2,737 patients with streptococcal BSIs from January 2010 to June 2020, analyzed the prevalence of IE in BSIs caused by different streptococcal species (Seo et al., 2023). The results showed that 12% of BSI cases caused by *S. oralis* (14/115) developed IE further confirming the significant association between IE and *S. oralis* BSIs (Seo et al., 2023). Taken together, these findings address the different distribution of streptococcal species in the development of IE and indicate that *S. oralis* is one of the high-risk species in streptococcal BSIs to cause IE. In this context, routine echocardiography has been recommended in patients with *S. oralis* BSIs.

### 2.2 Ocular infection

Infectious keratitis and endophthalmitis are rare but severe sight-threatening ocular infections (Simunovic et al., 2012; Gunalda et al., 2023). Without prompt and appropriate antimicrobial therapy, these diseases may result in irreversible visual loss (Gunalda et al., 2023). The common risk factors for infectious keratitis and endophthalmitis mainly include ocular trauma, ocular surgery, ocular surface disease, the use of contact lenses, and systemic immunodeficiency (Wong et al., 2012; Gunalda et al., 2023).

Following Staphylococcus spp., Streptococcus spp. is the second prevalent etiological agent in bacterial keratitis (Teweldemedhin et al., 2017). Compared with ocular infections caused by Staphylococcus spp., endophthalmitis and keratitis caused by streptococci usually progress more acutely and aggressively (Kuriyan et al., 2014). The prognoses of ocular infections caused by streptococci are thereby greatly compromised (Teweldemedhin et al., 2017; Santin et al., 2020). A recent study has demonstrated that *S. oralis* was the most prevalently identified causative agent in streptococcal endophthalmitis and keratitis, while *S. mitis* was only isolated in a small group of keratitis patients (Santin et al., 2020). This study analyzed the distribution of VGS species in endophthalmitis and keratitis by recovering 62 VGS isolates from patients with endophthalmitis (n=27; 2002-2013) and keratitis (n=35; 2009-2013) in Brazil. The results showed that the most predominately identified species was *S. oralis* accounting for 32.2% of cases (n=20), while *S. mitis* was only detected in 8.1% of cases (n=5). Even though early diagnosis and clinical therapy had been performed, the patients showed highly unfavorable visual acuity outcomes. One of the possible explanations is the high propensity of *S. oralis* to invade the posterior chamber (Santin et al., 2020).

Moreover, streptococci are also one of the leading causes of endophthalmitis following ocular interventions such as intravitreal injections and phakic intraocular lens (pIOL) implantations (Chen et al., 2011; Marquart et al., 2018; Busch et al., 2019; Cioana et al., 2024). An outbreak of streptococcal endophthalmitis caused by intravitreal injections of Bevacizumab has been reported in the USA (Matthews et al., 2014). In this outbreak, 10 out of 12 cases were caused by *S. mitis*/*oralis*. The pathogens have been demonstrated to originate from the contaminations during syringe preparation in pharmacy (Matthews et al., 2014). The majority of patients with *S. mitis*/*oralis* endophthalmitis showed poor visual outcomes where even an enucleation was sometimes required to remove infections (Matthews et al., 2014; Colás-Tomás and Pérez-Trigo, 2018). Additionally, it has been reported that *S. mitis*/*oralis* can also cause infectious endophthalmitis following pIOL implantation (Chung and Lee, 2014). Endophthalmitis in pIOL implantation is a relatively rare but potentially devastating complication. Thus, an early diagnosis is critically important. Compared with endophthalmitis caused by other pathogens, *S. mitis*/*oralis* endophthalmitis presents less virulent where the antibiotic treatment is effective without the removal of pIOL (Chung and Lee, 2014). However, the above-mentioned studies failed to differentiate between *S. mitis* and *S. oralis*. The distribution of *S. mitis* and *S. oralis* is required to be further addressed.

### 2.3 Meningitis

Bacterial meningitis is a global health menace with mortality rates ranging from 6% to 54% (Hasbun, 2022). It has been reported that the incidence of acute bacterial meningitis is 5–10/100,000 per year in developed countries, while the incidence in less developed countries is estimated even higher (Heckenberg et al., 2014). Given the high mortality of acute bacterial meningitis, a prompt diagnosis is paramount for tailoring appropriate antibiotic therapy and averting severe sequela such as permanent brain damage (Cruz Cardoso et al., 2021).

VGS species can cause meningitis in patients of all age groups. About 0.3–2.4% of meningitis cases are attributed to VGS (including *S. oralis*) (Montejo and Aguirrebengoe, 1998). In meningitis caused by VGS species, *S. oralis* is rarely implicated. To date, to our knowledge, about 10 cases of *S. oralis* meningitis have been reported in English-language literature (Fan et al., 2023). Although sporadic, *S. oralis* meningitis presents an association with oral diseases or oral manipulations. In several cases of *S. oralis* meningitis, it has been postulated that causative organisms originated from the oral cavity (Cruz Cardoso et al., 2021; Nakamura et al., 2021). Therefore, *S. oralis* meningitis should be taken into account when patients with oral diseases or dental procedure history present a fever, disturbance of consciousness, and headache (Nakamura et al., 2021). In other cases, *S. oralis* meningitis has been observed following spinal anesthesia for elective total knee replacement (Willder et al., 2013) and cerebrospinal fluid leaks (Patel et al., 2019). Additionally, *S. oralis* is also a rare causative agent of neonatal meningitis and maternal sepsis (Poi et al., 2018). In another case, meningoencephalitis and ventriculitis caused by *S. oralis* were reported in a 71-year-old female patient (Adly et al., 2023). However, risk factor from the oral cavity was ruled out in this case. *S. oralis* meningitis is also implicated in uncommon but severe complications such as cerebral vasospasm (Nonaka et al., 2018). Furthermore, a close scrutiny of the potential occurrence of IE in patients with meningitis associated with *S. oralis* is recommended (Patel et al., 2019; Cruz Cardoso et al., 2021).

### 2.4 Brain abscess

In addition to meningitis, *S. oralis* is also associated with brain abscesses (Solanki et al., 2014; Thiagarajan et al., 2016). Brain abscess is an intraparenchymal pyogenic infection with the reported incidence ranging from 0.98 to 1.28 per 100,000 population (Iro et al., 2023; Korkmaz and Korkmaz, 2023). It is a severe disease with a high potential of fatality (Muzumdar et al., 2011). The common predisposing factors mainly include an associated contiguous focus of infection, neurosurgery, head trauma, and hematogenous dissemination from a distant focus (Brook, 2017).

Brain abscesses caused by *S. oralis* are extremely uncommon. So far, to the best of our knowledge, two cases have been reported in the relevant English-language literature. In one case, *S. oralis* brain abscess was found in an infant bitten by a monkey wherein the pathogen might spread from the oral cavity of the monkey (Thiagarajan et al., 2016). In the other case, *S. oralis* brain abscess was observed in a 12-year-old patient with congenital heart disease (Solanki et al., 2014). In this case, congenital heart disease is assumed to be a putative predisposing factor in *S. oralis* infection while independent of oral hygiene (Solanki et al., 2014).

### 2.5 IE

IE is an infection in endocardium involving large intrathoracic vessels, native or prosthetic heart valves, or even cardiac chambers with substantial morbidity and mortality rates. As mentioned above, streptococcal BSI is one of the most dominant causes of IE (Chamat-Hedemand et al., 2020). A Spanish multicenter study has reported that VGS represented 27.5% of IE cases (Vicent et al., 2018). Among streptococcal species, *S. oralis* is the most common causative agent responsible for about 37.8% of streptococcal IE cases (Doyuk et al., 2002; Ercibengoa et al., 2019; Chamat-Hedemand et al., 2023a). Oral diseases such as caries and periodontitis, poor oral hygiene, and oral interventions are important risk factors in *S. oralis* endocarditis (Udayaraj et al., 2003; Nahhal et al., 2023). On the other hand, it has been surprisingly reported that *S. oralis* IE was developed in an edentulous patient without any predisposing conditions such as underlying valvular heart disease, systemic infections at other sites, or dental procedure history (Renton et al., 2009). The mortality and therapy could differ remarkably from streptococcal species. For example, IE caused by *S. oralis* requires heart valve surgery more frequently than that caused by *Streptococcus gallolyticus* (Chamat-Hedemand et al., 2023a). It is postulated that *S. oralis* can grow more in plasma or thrombotic vegetation compared with other oral streptococcal species (Doyuk et al., 2002; Nagata et al., 2005). Therefore, an accurate and expedited diagnosis is vitally important in the treatment of IE. However, in clinical practice, the etiologic agent of IE has been predominantly identified at the group level, whilst the species-level classification has been seldom performed (Chamat-Hedemand et al., 2023a).

In high-income countries, the epidemiology of IE presents a shift from occurring in native valves to occurring in prosthetic valves or implantable cardiovascular devices of elderly patients (Hill et al., 2007). In addition to native valve endocarditis, *S.oralis* has also been recognized in prosthetic valve endocarditis (Turnier et al., 2009). The prevalence of *S. oralis* among patients with native valve endocarditis is 7%, while the prevalence among patients with prosthetic valve endocarditis is 5% (Chamat-Hedemand et al., 2020). The beta-lactam-resistant *S. oralis* has emerged in prosthetic valve IE imposing formidable clinical challenges (Tanaka et al., 2022). In this case, a patient who had native valve IE caused by beta-lactam-susceptible *S. oralis* has undergone prosthetic valve replacement (Tanaka et al., 2022). Subsequently, prosthetic valve IE caused by highly beta-lactam-resistant *S. oralis* was developed. This case confirms the evolution of antibiotic resistance in *S. oralis* and reflects the importance of antimicrobial therapy based on the susceptibility of specific species.

Notably, *S. oralis* has also been identified in uncommon IE cases such as pulmonic valve endocarditis and IE in pregnancy. Right-sided endocarditis occurs less frequently than left-sided endocarditis accounting for approximately 10% of IE cases (Shmueli et al., 2020). Furthermore, right-sided endocarditis largely involves the tricuspid valve but rarely involves the pulmonic valve (Fishbein and Fishbein, 2019). *S. oralis* has been recognized as a causative agent in pulmonic valve endocarditis (Goud et al., 2015; Nahhal et al., 2023). In one case, pulmonic valve endocarditis was caused by *S. oralis* BSI secondary to a dental abscess (Nahhal et al., 2023). IE in pregnancy is also uncommon with an estimated incidence of 1 per 100,000 per year (Montoya et al., 2003). Nonetheless, IE in pregnancy is devasting with maternal and fetal mortalities of 22.1% and 14.7%, respectively (Yuan, 2015). Wydall et al. (2021) identified *S. oralis* as the causative microorganism for IE and bacterial meningitis in a pregnant patient.

In the pathogenesis of IE, binding to platelets is a crucial step (Werdan et al., 2014). Previous work has identified several virulence factors such as serine-rich repeat protein (SRRP), associated with sialic acid adhesion A (AsaA), and neuraminidases A (NanA) in *S. oralis* IE isolates (Singh et al., 2017; Ronis et al., 2019; Gaytán et al., 2021). Additionally, it has been demonstrated that these virulence factors play a crucial role in regulating *S. oralis* adherence to platelets by binding to sialic acid (Singh et al., 2017; Ronis et al., 2019; Gaytán et al., 2021). Furthermore, Gaytán et al. (2021) have observed a significant reduction in colony-forming units (CFUs) in the rabbits with aortic valve damage inoculated with the *asaA* mutant compared with the parent strain. The evidence collectively indicates the pathogenic potential of these virulence factors in the development of IE caused by *S.oralis*.

### 2.6 Peritonitis

Peritoneal dialysis (PD)-related peritonitis is the most common and severe complication of PD resulting in PD catheter removal transition to hemodialysis, encapsulation peritoneal sclerosis, and mortality. About 5-10% of all cases of PD-related peritonitis are attributed to VGS species (Chao et al., 2015; Liu et al., 2018). Nonetheless, the information concerning the identification and distribution of different VGS species is extremely scarce. To date, few cases of PD-related peritonitis have been reported to be caused by *S. oralis*. Two of them assumed that the pathogen originated from the oral cavity via hematogenous spread (Koruk et al., 2005; Mihara et al., 2023), while the other two cases excluded the oral entry route (Kotani et al., 2021). Favorable clinical outcomes have been observed in patients with PD-related peritonitis caused by *S. oralis*, and the outcomes do not differ from VGS species (Liu et al., 2018; Kotani et al., 2021).

### 2.7 Urinary tract infection

UTI is one of the most common infectious diseases in clinical practice owing to the anatomic features of the human urinary tract (Foxman, 2010). It is estimated that approximately 150 million people are affected by UTIs every year worldwide (Flores-Mireles et al., 2015). Albeit the low morbidity, the high incidence of UTIs causes a substantial society economic burden by costing over US $3.5 billion each year in the USA alone (Neugent et al., 2020). Predominant UTI pathogens include *Escherichia coli*, *Klebsiella pneumoniae*, *Proteus mirabilis*, *Enterococcus faecalis*, and *Staphylococcus saprophyticus*. The identification of *S. mitis*/*oralis* in urine has been taken as commensals or contamination leading to an underestimation of UTIs associated with this species (Nelson et al., 2010; Mores et al., 2021). Nevertheless, growing case reports have revealed that *S. mitis*/*oralis* could cause UTIs in both adults and children with compromised immune systems due to alcoholic liver disease, renal transplant, and diabetes (Ong et al., 1998; Swain and Otta, 2013; Zhang et al., 2023). Furthermore, *S. mitis*/*oralis* has emerged as multidrug-resistant rendering antibiotic therapy difficult (Zhang et al., 2023). However, previous research failed to differentiate between *S. mitis* and *S. oralis*. The species-level identification is recommended for the determination of appropriate antibiotic regimens and understanding of the epidemiology of *S. oralis* UTIs.

### 2.8 Spinal abscess

Pyogenic spinal infection is a rare but highly fatal disease with a mortality rate of 2-20% in developed countries (Lener et al., 2018). Common predisposing factors for pyogenic spinal infection encompass immunodeficiency, intravenous drug use, and spine procedures (Schwab and Shah, 2020). *S. oralis* can be transmitted hematogenously to the brain, spinal cord, and spine due to spinal instrumentation, anesthesia, or oral infectious diseases (Willder et al., 2013; Prod’homme et al., 2021). It has been reported that *S. oralis* led to spinal abscesses in immunocompromised patients (diabetes) (Chu et al., 2022). In this case, the patient presented gingivitis. It has been speculated that *S. oralis* was spread hematogenously from gingivitis to the spine.

## 3 Conclusions

Molecular techniques have illuminated the role of *S. oralis* as one of the major causative agents among VGS infections and suggested variations in distribution, clinical features, and prognosis within VGS (Kitten et al., 2012; Sahasrabhojane et al., 2014; Chun et al., 2015; Kim et al., 2018). The present study has offered a concise summary of *S. oralis* opportunistic infections (**Table 1**). The insights gained here may be of assistance to the correct diagnosis and optimizing antimicrobial therapy of relative infectious diseases in clinical practice. Although the role of *S. oralis* as an opportunistic pathogen has been uncovered, the epidemiology of *S. oralis* infections is poorly understood owing to the lack of species-level identification of VGS, especially the correct differentiation between *S. mitis* and *S. oralis*. Therefore, on the one hand, future investigations are required to provide a simple, precise, and reliable assignment of *S. oralis*. Additionally, clinicians should pay more attention to *S. oralis* opportunistic infections to further clarify the epidemiology of *S. oralis* infectious diseases.

**Table 1:** *S. oralis* opportunistic infections.

Infection	References	Study description
Bloodstream infection	(Kennedy et al., 2000; Kennedy et al., 2003; Shelburne et al., 2014; Basaranoglu et al., 2019; Chamat-Hedemand et al., 2020; Watanabe et al., 2020; Chamat-Hedemand et al., 2023b; Seo et al., 2023)	Shelburne et al., 2014, cohort study of patients with VGS BSIs between July 1, 2011, and December 1, 2012 (*S. oralis* n=22) Chamat-Hedemand et al., 2023b, cohort study of patients with streptococcal BSIs between January 1, 2008, and December 31, 2017 (*S. mitis*/*oralis* n = 385) Kennedy et al., 2000, case report of polymicrobial BSI caused by *Staphylococcus epidermidis* and *S. oralis* in a 15-year-old boy following bone marrow transplantation in 1999 Basaranoglu et al., 2019, case-control study of *S. mitis*/*oralis* BSIs in pediatric patients between January 2015 and March 2017 (*S. mitis*/*oralis* n=53) Kennedy et al., 2003, case report of *S. oralis* BSI in a 12-year-old neutropenic boy with severe gingivitis in 2002 Watanabe et al., 2020, case report of multidrug-resistant *S. oralis* BSI in a 30-year-old female patient with leukemia in 2019 Chamat-Hedemand et al., 2020, cohort study of IE prevalence at species level in patients with streptococcal BSIs from 2008 to 2017 (*S. mitis*/*oralis* n=408) Seo et al., 2023, cohort study of patients with streptococcal BSIs between January 2010 and June 2020 (*S. oralis* n=115)
Ocular infection	(Chung and Lee, 2014; Matthews et al., 2014; Colás-Tomás and Pérez-Trigo, 2018; Marquart et al., 2018; Busch et al., 2019; Santin et al., 2020)	Santin et al., 2020, cohort study of 27 and 35 alpha-hemolytic streptococci isolates recovered from patients with infectious 13 endophthalmitis (2002-2013) and 7 keratitis (2008-2013) (*S. oralis* n=20) Marquart et al., 2018, cohort study of 22 VGS endophthalmitis strains (*S. mitis*/*oralis* n=15) Busch et al., 2019, cohort study of patients with culture-positive endophthalmitis after intravitreal anti-VEGF injection from January 1, 2011, to December 31, 2016 (*S. oralis* n=1) Matthews et al., 2014, case series of an outbreak of *S. mitis*/*oralis* endophthalmitis between July 5 and July 8, 2011 (*S. mitis*/*oralis* n=10) Colás-Tomás and Pérez-Trigo, 2018, case report of *S. oralis* endophthalmitis following implantation in a 54-year-old male patient in 2018 Chung and Lee, 2014, case report of *S. mitis*/*oralis* endophthalmitis following pIOL implantation in a 23-year-old woman in 2014
Meningitis	(Montejo and Aguirrebengoe, 1998; Willder et al., 2013; Nonaka et al., 2018; Poi et al., 2018; Patel et al., 2019; Cruz Cardoso et al., 2021; Nakamura et al., 2021; Adly et al., 2023; Fan et al., 2023)	Cruz Cardoso et al., 2021, case report of *S. oralis* meningitis in a 53-year-old man with poor oral hygiene in 2021 Montejo and Aguirrebengoe, 1998, case report of *S. oralis* meningitis associated with dental extraction in a 48-year-old woman in 1998 Fan et al., 2023, case report of *S. oralis* meningitis in a 71-year-old man with gingivitis in 2023 Nakamura et al., 2021, case report of *S. oralis* meningitis in an 81-year-old male patient with gingival bleeding in 2020 Willder et al., 2013, case report of *S. oralis* meningitis following spinal anesthesia for elective total knee replacement in an 81-year-old woman in 2013 Patel et al., 2019, case report of *S. oralis* meningitis in a 58-year-old patient with cerebral spinal fluid leak in 2019 Poi et al., 2018, case report of neonatal meningitis and maternal sepsis caused by *S. oralis* in 2018 Adly et al., 2023, case report of *S. oralis*-induced meningoencephalitis and ventriculitis in a 71-year-old female geriatric patient in 2023 Nonaka et al., 2018, case report of cerebral vasospasm secondary to *S. oralis* meningitis in a 37-year-old female patient in 2018
Brain abscess	(Solanki et al., 2014; Thiagarajan et al., 2016)	Thiagarajan et al., 2016, case report of *S. oralis* cerebral abscess following monkey bite in a 2-month-old infant in 2015 Solanki et al., 2014, case report of *S. oralis* brain abscess in a 12-year-old girl with congenital heart disease in 2013
Infective endocarditis	(Doyuk et al., 2002; Udayaraj et al., 2003; Renton et al., 2009; Turnier et al., 2009; Goud et al., 2015; Ercibengoa et al., 2019; Chamat-Hedemand et al., 2020; Wydall et al., 2021; Tanaka et al., 2022; Chamat-Hedemand et al., 2023a; Nahhal et al., 2023)	Chamat-Hedemand et al., 2020, cohort study of IE prevalence at species level in patients with streptococcal BSIs from 2008 to 2017 (*S. mitis*/*oralis* n=408) Chamat-Hedemand et al., 2023a, cohort study of patients with streptococcal IE from October 1, 2002, to October 31, 2012 (*S. mitis/oralis* n=60) Doyuk et al., 2002, case report of polymicrobial IE caused by *Streptococcus vestibularis* and *S. oralis* in a 73-year-old female patient in 2002 Ercibengoa et al., 2019, cohort study of f patients with streptococcal IE between 2008 and 2016 (*S. oralis* n = 28) Nahhal et al., 2023, case report of *S. oralis* pulmonic valve endocarditis in an 81-year-old male patient in 2023 Udayaraj et al., 2003, case report of septic discitis as a complication of *S. oralis* IE in a 60-year-old male patient in 2002 Renton et al., 2009, case report of *S. oralis* endocarditis in a 70-year-old female edentulous patient in 2008 Turnier et al., 2009, case report of fatal aortic valve prosthetic valve endocarditis caused by *S. oralis* in a 42-year-old woman in 2008 Tanaka et al., 2022, case report of prosthetic valve endocarditis due to highly beta-lactam-resistant *S. oralis* in a 79-year-old male patient in 2022 Goud et al., 2015, case report of cervical discitis and pulmonic valve endocarditis caused by *S. oralis* in a 52-year-old male patient in 2015 Wydall et al., 2021, case report of *S. oralis* endocarditis leading to central nervous system infection in pregnancy in 2021
Peritonitis	(Koruk et al., 2005; Kotani et al., 2021; Mihara et al., 2023)	Koruk et al., 2005, case report of *S. oralis* peritonitis in a 40-year-old woman undergoing continuous ambulatory peritoneal dialysis in 2005 Mihara et al., 2023, case report of *S. oralis* peritonitis in a 60-year-old male patient with end-stage renal disease in 2023 Kotani et al., 2021, case report of *S. oralis* peritonitis in a 77-year-old male patient without major dental disease or recent history of dental intervention in 2021
Urinary tract infection	(Ong et al., 1998; Swain and Otta, 2013; Zhang et al., 2023)	Zhang et al., 2023, case report of urinary tract infection caused by multidrug-resistant *S. mitis/oralis* in a 66-year-old male patient in 2023 Ong et al., 1998), case report of *S. mitis*/*oralis* UTI in an 11-year-old male renal transplant patient in 1997 Swain and Otta, 2013, case report of *S. mitis*/*oralis* UTI in a 55-year-old female diabetic patient in 2013
Spinal abscess	(Prod’homme et al., 2021; Chu et al., 2022)	Prod’homme et al., 2021, case report of epidural abscess related to *S. mitis*/*oralis* in an immunocompetent 57-year-old man in 2021 Chu et al., 2022, case report of spinal abscess due to *S. oralis* in a 60-year-old man with gingivitis and poorly controlled diabetes in 2022

In addition to the epidemiology, the pathogenesis of *S. oralis* also remains elusive. On the one hand, many previous studies failed to illustrate the entry route of *S. oralis* in infectious diseases. The association between the oral entry route and *S. oralis* infections needs to be explored. Another potentially fruitful avenue for future research is the pathogenic potential of *S. oralis*. It has been established that as one of the closest relatives of *S. pneumoniae*, *S. oralis* shares a variety of common virulence factors with *S. pneumoniae* such as choline-binding proteins, neuraminidases A, immunoglobulin A1 proteases, and zinc metalloproteases (Kilian and Tettelin, 2019). Moreover, the interspecies gene transfer between *S. pneumoniae* and *S. oralis* allows the emergence of more common virulence factors (Joyce et al., 2022). Owing to the role of *S. pneumoniae* as a major human pathogen, the functions of these virulence factors involving the adhesion and invasiveness of *S. pneumonia* have been extensively studied (Sadowy and Hryniewicz, 2020). By contrast, the exact functions of these virulence factors in the pathogenesis of *S. oralis* infections are still unexplored. Particularly very little is currently known about the underlying pathogenic mechanisms that trigger the alteration from commensals to opportunistic pathogens of *S. oralis*. Further research on this topic is needed to develop a full picture of *S. oralis* opportunistic infections.
